# Molecular Insights into Epigenetics and Cannabinoid Receptors

**DOI:** 10.3390/biom12111560

**Published:** 2022-10-26

**Authors:** Balapal S. Basavarajappa, Shivakumar Subbanna

**Affiliations:** 1 Center for Dementia Research, Nathan Kline Institute for Psychiatric Research, Orangeburg, NY 10962, USA; 2 Molecular Imaging and Neuropathology Area, New York State Psychiatric Institute, New York, NY 10032, USA; 3 Department of Psychiatry, Columbia University Irving Medical Center, New York, NY 10032, USA; 4 Department of Psychiatry, New York University Langone Medical Center, New York, NY 10016, USA

**Keywords:** cannabinoids, histone, DNA, methylation, microRNA, acetylation, synaptic plasticity, learning and memory, cognitive behavior, intellectual disabilities, drugs of abuse

## Abstract

The actions of cannabis are mediated by G protein-coupled receptors that are part of an endogenous cannabinoid system (ECS). ECS consists of the naturally occurring ligands N-arachidonylethanolamine (anandamide) and 2-arachidonoylglycerol (2-AG), their biosynthetic and degradative enzymes, and the CB_1_ and CB_2_ cannabinoid receptors. Epigenetics are heritable changes that affect gene expression without changing the DNA sequence, transducing external stimuli in stable alterations of the DNA or chromatin structure. Cannabinoid receptors are crucial candidates for exploring their functions through epigenetic approaches due to their significant roles in health and diseases. Epigenetic changes usually promote alterations in the expression of genes and proteins that can be evaluated by various transcriptomic and proteomic analyses. Despite the exponential growth of new evidence on the critical functions of cannabinoid receptors, much is still unknown regarding the contribution of various genetic and epigenetic factors that regulate cannabinoid receptor gene expression. Recent studies have identified several immediate and long-lasting epigenetic changes, such as DNA methylation, DNA-associated histone proteins, and RNA regulatory networks, in cannabinoid receptor function. Thus, they can offer solutions to many cellular, molecular, and behavioral impairments found after modulation of cannabinoid receptor activities. In this review, we discuss the significant research advances in different epigenetic factors contributing to the regulation of cannabinoid receptors and their functions under both physiological and pathological conditions. Increasing our understanding of the epigenetics of cannabinoid receptors will significantly advance our knowledge and could lead to the identification of novel therapeutic targets and innovative treatment strategies for diseases associated with altered cannabinoid receptor functions.

## 1. Introduction

### 1.1. Endocannabinoid System: A Brief Overview

Endocannabinoids (eCBs) are bioactive lipids implicated in many physiological mechanisms in the central nervous system (CNS) and peripheral tissues. The eCBs N-arachidonylethanolamine (anandamide, AEA) and 2-arachidonoylglycerol (2-AG) are primarily synthesized in all cell types because they are derived from phospholipids containing arachidonic acid (AA) [1,2,3,4]. The anandamide-synthesizing enzyme is N-acylphosphatidylethanolamine (NAPE)-phospholipase D hydrolase (NAPE-PLD), which catalyzes the synthesis of AEA [5]. Diacylglycerol lipase (DAGL) catalyzes the biosynthesis of 2-AG [6]. In addition to AEA and 2-AG synthesis, other N-acylethanolamines, such as oleoylethanolamide (OEA), linoleoylethanolamide (LEA), palmitoylethanolamide (PEA), and docosahexaenoylethanolamine (DHEA), and other 2-acylglycerols, such as 2-oleoylglycerol and 2-linoleoylglycerol, are synthesized through alternative biochemical routes [7]. These bioactive lipids exhibit different affinities at the cannabinoid receptors CB_1_ and CB_2_ and other receptors (transient receptor potential vanilloid 1 (TRPV1), peroxisome proliferator-activated nuclear receptor-α (PPARα) and PPARγ, and the orphan G protein-coupled receptors GPR55 and GPR119 [8]). Upon their action at the CB receptors, eCBs undergo rapid degradation by fatty acid amide hydrolase (FAAH) [9] and monoacylglycerol lipase (MAGL) [10] to ethanolamine, glycerol, AA, and other fatty acids [11,12]. Details on eCB, biosynthetic, and metabolism pathways have been extensively reviewed in recent publications [7,13].

CB_1_ and CB_2_ are the extensively studied receptor targets of eCBs, which bind to and activate them with different affinities. CB_1_ is the brain’s most abundant G protein-coupled receptor [9]. It is responsible for mediating most of the neurobehavioral effects of Δ^9^ -tetrahydrocannabinol (THC) [14,15], the psychoactive constituent of marijuana [16,17]. Consistent with the well-established functions of eCB, CB_1_ is enriched in brain areas implicated in memory (e.g., hippocampus, HP), motor coordination (e.g., basal ganglia, cerebellum), and emotional processes (e.g., prefrontal cortex, PFC; amygdala, Amy) [18,19]. CB_1_ is preferentially restricted to the presynaptic region, and eCBs released from postsynaptic neurons act retrogradely on presynaptic CB_1_, resulting in short- and long-term suppression of neurotransmitter release [20,21] and the modulation of neuronal activity and network function. This intricate circuit influences various pathophysiological functions, such as emotion, cognition, energy balance, pain sensation, and neuroinflammation [7]. CB_1_ is also expressed in peripheral tissues, including adipose tissue, liver, and skeletal muscle [22]. CB_2_ is predominantly expressed in immune cells [23,24], where it seems to facilitate the immunosuppressive effects of eCBs. Interestingly, further discoveries emphasize that CB_2_ is expressed at low levels in some areas of the brain [24,25,26], where it is activated during injury and inflammation [25,26]. CB_2_ is mostly located in postsynaptic terminals [27]. However, CB_2_ is also expressed in some presynaptic terminals [26,28,29].

CB_1_ is encoded by the CNR1 gene and comprises 472 amino acids in humans and 473 amino acids in rodents (rats and mice), with 97–99% amino acid sequence similarity among them [30]. The CNR1 gene is localized to human chromosome 6q14–15 and mouse chromosome 4. Human CNR1 has four exons, with exon 4 containing the entire protein-coding region [31]. In mice and rats, the coding region of CNR1 is contained within a single exon. Although the 5′ untranslated regions (5′-UTRs) and promoter structures differ between mice and humans [32,33], these structures are not well described in rats [34]. The longest 5′ UTR in human CNR1 is approximately 500 nucleotides and has approximately 600 potential transcription factor-binding regions for 153 distinct transcription factors [35,36]. A few of these binding sites are unique and bind signal transducer and activator of transcription proteins and eventually may regulate many critical aspects of cell growth, survival, and differentiation [36]. The complexity of 5′ UTR of the CNR1 gene emphasizes that multiple transcription factors regulate CNR1 gene expression in a basic manner, which may be necessary for brain development and function [36]. A polymorphic enhancer sequence (ECR1) was identified within intron 2 of the CNR1 locus [37], and disruption of ECR1 using CRISPR genome editing in mice indicated that ECR1 is essential for maintaining normal levels of CNR1 expression within the hippocampus [38]. The CB_1_ mRNA distribution also paralleled that of the CB_1_ protein in certain brain areas [39,40,41].

CB_2_ is encoded by the CNR2 gene and is located on human chromosome 1p36 and mouse chromosome 4 [42,43,44,45]. CB_2_ displays less homology between species than CB_1_; for example, human and mouse CB_2_ share an 82% amino acid homology [46], and mouse and rat CB_2_ share a 93% amino acid homology. The human, rat, and mouse sequences differ at the C-terminus [47]. The mouse sequence is 13 amino acids shorter, whereas the rat clone is 50 amino acids longer than human CB_2_ [47]. The CNR2 mRNA distribution has been detected in multiple brain regions, including the PFC, hippocampus, midbrain, and cerebellum [48,49,50,51]. The human CNR2 gene has three exons with three separate promoters [52,53]. However, the current evidence indicates significant species differences in CNR2s in humans, mice, and rats regarding mRNA sizes and gene structure [49,52,53]. Although the functional implication of multiple transcription start sites (TSSs) and core promoters is unknown, this heterogeneity may have significance for the cell type and activation function [54,55]. Additionally, the promoter region of human CNR2 has several transcription factor-binding sites [56,57], which can regulate the expression of CB_2_ [57]. The promoter of CNR2 has cytosine-phosphate-guanine (CpG) islands and many CCAAT boxes with binding sites for transcription factors associated with the stress response, such as activator protein-1 (AP1), heat shock factor (HSF) and stress response element, GATA-binding factor-1 (erythroid transcription factor), tinman homolog Ntx2.5 (homeodomain factor), and AP4 [58]. The epigenetic regulation of CNR2 loci via DNA methylation might play a decisive function in receptor regulation due to the CpG islands found in the promoter regions. These gene regulatory binding sites are significant, as recent studies have indicated that cannabinoid receptor gene expression could be controlled by chemical modification of DNA and histone tails (epigenetics), resulting in alterations in the chromatin structure and access to transcription factors.

### 1.2. Epigenetic Mechanisms: A Brief Overview

In general, the primary epigenetic mechanisms that are well recognized to control the expression of genes in the CNS are (1) the main chemical modification of DNA through methylation (-CH_3_) of cytosine residues in promoter-rich CpG islands; (2) the acetylation (ac), mono- (me1), di- (me2), and tri-methylation (me3) at lysine (K) residues, and other covalent post-translational modification (PTM) of DNA-associated histone protein tails; (3) chromatin remodeling factors that affect gene transcription; (4) the editing and splicing of pre-mRNA by noncoding, small nucleolar RNAs (snoRNAs); e) microRNAs (miRNAs), mRNA processing, the translation and stability of binding proteins, and long noncoding RNAs (lncRNAs); and (5) cellular signaling molecules controlling mRNA translation. Recent publications have reviewed the fundamental features of these epigenetic processes in detail [59,60,61,62,63]. These epigenetic factors/regulators can selectively respond to adverse environmental conditions, causing alterations in the brain’s physiological function and pathological processes. An increasing number of adverse conditions, including exposure to cannabinoids, which activate cannabinoid receptors, have undoubtedly been shown to alter various epigenetic factors. Nevertheless, the mechanism by which altered epigenetic events cause cannabinoid receptor- or cannabinoid-mediated gene expression is poorly defined. In this review, we provide the current understanding of epigenetic changes in cannabinoid receptor gene regulatory regions and cannabinoid-mediated events.

## 2. Role of DNA Methylation: Cannabinoid Receptors

DNA methylation and other remodeling factors are considered significant epigenetic markers and are known to control gene expression (for reference, see [64,65]). DNA de novo methylation, which occurs in distinct cellular contexts in germ cells and during maturation, is catalyzed by DNA methyltransferases 3A (DNMT3A) and 3B (DNMT3B) in partnership with DNMT3L, a DNMT devoid of catalytic activity. However, it facilitates de novo methylation by promoting the ability of DNMTs to bind to the methyl group donor S-adenosyl-L-methionine (SAM). Additionally, DNA methylation is stabilized by DNMT1. Studies have suggested at least two routes through which the DNA demethylation process occurs: (1) deaminase activity catalyzes the conversion of methylcytosine (mC) to thymidine [66] and (2) the action of the ten-eleven translocation (*TET*) family (α-ketoglutarate-dependent dioxygenases). TET proteins oxidize 5-mC to 5-hydroxymethylcytosine (5-hmC) using oxygen- and α-ketoglutarate-dependent pathways [67]. DNA demethylation processes through 5-hmC were revealed to function in both developing and adult brains [68], thereby offering the basis for a valuable epigenetic regulator of gene expression [69].

In addition, a different group of proteins that work together with methylated DNA to control gene expression in CNS is the family of methyl CpG-binding proteins (MeCPs). Methyl CpG-binding proteins often function as gene suppressors by binding to methylated cytosines [70,71] in DNA. The MeCP2 protein recognizes and binds to single methylated cytosine (5mC) sites in DNA. Additionally, the binding of MeCP2 to DNA further facilitates the recruitment of transcriptional corepressor complexes [70]. Moreover, phosphorylation of MeCP2 affects its capacity to bind to DNA and regulate gene expression [72,73]. The activity-dependent phosphorylation of MeCP2 promotes its dissociation from promoters, thereby facilitating the DNA demethylation process. Thus, DNA methylation followed by the binding of MeCP2 appears to have a central role in gene expression.

### 2.1. DNA Methylation on CB_1_ Receptor Gene (Cnr1) Expression

In the past decade, evidence has accumulated suggesting that CB_1_ gene expression is under the control of epigenetic mechanisms. This is partly because CB_1_ gene expression is altered in response to different pathological conditions and upon exposure to adverse insults, including exposure to different drugs [74]. Additionally, many transcription factors implicated in DNA methylation and histone post-translational modifications interact with cannabinoid receptor genes [36,56,57]. The first study demonstrating the association of DNA hypermethylation of the CNR1 gene promoter contributing to the downregulation of CNR1 gene transcription was observed in colon cancer specimens [75]. A similar observation was found in another study in which exposure to prostaglandin E2 suppressed CNR1 gene expression by increasing DNA methylation in the CNR1 promoter region in the human epithelial colon cell line LS-174T, causing tumor growth [76]. Enhanced DNA methylation in the Cnr1 gene promoter region was also found in rodents after maternal separation from postnatal day (PD) 1 to 14 in the first-generation germline [77]. This outcome supports the previously well-established function of CB_1_ in emotional behavior [78]. In another study, although the mechanisms are less clear, the expression of CB_1_ by an inhibitor of DNA methyltransferases (5-aza-2′-deoxycytidine, 5-Aza-dC) was found only in those cells (Jurkat cells) in which the expression of CB_1_ was constitutively inactive [79]. In another study, it was found that DNA hypermethylation of the CNR1 gene promoter was associated with reduced CNR1 mRNA levels in peripheral blood cells of subjects with THC dependence [80]. Selective and transient upregulation of CNR1 gene expression was observed in human colon cancer cells (Caco-2) and rats exposed to short- and long-term dietary extra-virgin olive oil (EVOO) and its phenolic extracts (OPE) or authentic hydroxytyrosol (HT) [81]. Additionally, this treatment caused a reduction in DNA methylation at the Cnr1 gene promoter [81]. Chronic stress-induced visceral pain in the peripheral nervous systems of rats was associated with enhanced DNMT1-mediated DNA hypermethylation at the Cnr1 gene promoter [82]. Furthermore, it decreased Cnr1 gene expression in L6-S2 that transmit pain (nociceptive) signals but not L4-L5 dorsal root ganglia (DRG) [82].

Similarly, enhanced Cnr1 gene expression in PFC was associated with reduced DNA methylation at the Cnr1 gene promoter in a well-validated animal model of schizophrenia (prenatal methylazoxymethanol acetate exposure in rats) and in schizophrenic patients [83]. A significant and selective increase in Cnr1 gene expression in the hypothalamus (HTM) was observed in the initial stages of obesity onset (5 weeks on a high-fat diet) and after 21 weeks of high-fat diet consumption. In addition, there was a significant reduction in DNA methylation at specific CpG sites at Cnr1 gene promoters [84]. Similar observations were found in blood mononuclear cells from younger (<30 years old) human obese subjects [84]. Exposure to THC or alcohol is significantly associated with increased expression of CNR1 in PFC of patients with affective disorder [36]. Additionally, enhanced CNR1 expression was observed in PFC of schizophrenia patients who had committed suicide [36]. It was found that DNA methylation (cg02498983 allele, associated with CNR1 expression) is inversely associated with CNR1 expression [36]. In an activity-based anorexia rat model, Cnr1 gene expression was associated with significant increases in DNA methylation at the Cnr1 gene promoter in the HTM and nucleus accumbens (NAc) brain regions [85]. 

In an animal model of eating addictive-like behavior, a significant loss of DNA methylation at the Cnr1 gene promoter was observed in PFC. This loss was associated with enhanced CB_1_ protein expression in the same brain area [86]. Additionally, the pharmacological blockade of CB_1_ activity during the late training period significantly impaired addictive behavior in mice [86]. This latter observation agreed with the impaired performance of CB_1_-null mice in this operant training [86]. These findings suggest that DNA methylation-mediated CB_1_ expression could influence addictive behavior. In another study, selective reduction of DNA methylation at the promoter of CNR1 and enhanced CNR1 gene expression were observed in schizophrenic patients, with no changes in any other disorder [83]. These results from different experimental models indicate that DNA methylation events regulate Cnr1 gene expression (Table 1).

### 2.2. DNA Methylation on CB_2_ Gene (Cnr2) Expression

Compared to Cnr1, Cnr2 gene regulation by DNA methylation mechanisms has been less studied. However, THC consumption has been shown to enhance CB_2_ expression in human blood lymphocytes via changes in DNMT and TET mRNAs [89]. Although no direct link between these events was established, these observations may suggest that increased DNMT-methylating enzymes are associated with some of the pathophysiological processes in schizophrenia and, therefore, should be one of the potential mechanisms linking cannabis use as a trigger for schizophrenia in vulnerable individuals. In another study, CB_2_-selective agonist (JWH-133)-treated male mice crossed with untreated females exhibited embryonic and placental defects. Additional analysis indicated significantly reduced Tet3 expression in sperm. In addition, significantly increased enrichment of 5mC and reduced 5hmC at paternally expressed genes (*Peg10* and *Plagl1)* in the sperm of JWH-133-treated males was found [90]. In another study, the expression of CB_2_ by an inhibitor of DNA methyltransferases, 5-Aza-dC, was found only in cells where the expression of the CB_2_ receptor was silenced. Thus, CB2 was induced by 5-Aza-dC only in SH SY5Y cells but not in Jurkat cells. Although the mechanism is unclear, these findings suggest that already constitutively expressed genes were not regulated in these cells. Altogether, these limited studies suggest that CB_2_ expression could be regulated by DNA methylation of its promoter and warrant future studies, especially during inflammation [91,92,93], fear memory [94], nerve injury [52], and compulsive drug abuse [95] conditions in which CB_2_ expression was found to be heightened.

### 2.3. Cannabinoid Receptor Stimulation on DNA Methylation

Several studies using eCBs or agonists or antagonists acting specifically through CB_1_ have also demonstrated the participation of DNA methylation in several biological functions. For example, in human keratinocytes (HaCaT cells), AEA reduced *keratin 1*, *keratin 10*, *involucrin*, and *transglutaminase-5* gene expression by DNA hypermethylation. Treatment of HaCaT cells with 5-azacytidine ameliorated AEA-inhibited *keratin* gene expression, indicating that AEA itself was also able to suppress gene transcription by altering both specific and global DNA methylation [96]. Furthermore, it was found that AEA-induced DNMT activity in differentiated keratinocytes was CB_1_ dependent via p38 MAPK signaling [96]. In THC-treated SIV-infected macaques, it was found that hypermethylation of DNA of several genes was critical for the replication and pathogenesis of human (HIV) and simian (SIV) immunodeficiency viruses [97]. These findings indicated that eCBs could function as transcriptional repressors via less defined DNA methylation mechanisms. In another study, exposure to the CB_1_ agonist WIN55,212-2 during adolescence increased DNMT3a expression and inhibited cocaine-induced conditioned place preference in mice [98]. THC administration via oral gavage in rats caused significant hypermethylation at *Lrrtm4* and significant hypomethylation at *Shank1*, *Syt3*, *Nrxn1*, *Nrxn3*, *Dlg4*, and *Grid1* of neurodevelopmental genes in rat sperm [99]. THC consumption in patients who have schizophrenic psychosis caused high DNA methylation at the NEUREXIN (NRXN1) promoter, a schizophrenia candidate gene, compared to controls and non-THC consumer patients [100]. Administration of WIN55,212-2 to adolescent rats induced DNA hypermethylation at the intragenic region of the *Rgs7* gene, which was associated with a lower rate of mRNA transcription of the *Rgs7* gene [101]. Rgs7 acts as an intracellular antagonist of GPCR signaling [101]. It was shown that reduced expression of cannabinoid receptor-interacting protein 1 (CNRIP1) was associated with enhanced DNA methylation of a CpG island site named CNRIP1 MS-2 (CNRIP1 methylation site-2) in intrahepatic cholangiocarcinoma (ICC) cells [102].

Alcohol exposure during development can affect brain development and cause persistent behavioral problems. For example, exposure of PD-7 mice to alcohol heightened CB_1_ activity (enhanced CB_1_ expression and anandamide levels) and caused neurodegeneration as measured by active caspase-3 levels [87]. In the same animal model, PD-7 alcohol exposure reduced global DNA methylation by promoting the loss of DNMT1 and DNMT3A in the neonatal brain, and these losses were not observed in CB_1_-null mice [88]. Additionally, blockade of CB_1_ with antagonist (SR141716A) prior to PD-7 alcohol exposure in wild-type mice also prevented the loss of DNA methylation [88]. These findings suggest the potential of CB_1_ in regulating the DNA methylation process. In addition, reduced MeCP2, a protein essential for synaptogenesis and neuronal maturation, was observed in these conditions [103]. Interestingly, the genetic deletion of CB_1_ prevented the loss of the MeCP2 protein in alcohol-exposed PD-7 mice, and administration of a CB_1_ antagonist (SR141716A) before PD-7 alcohol exposure precluded this loss [103]. These observations suggested that CB_1_-mediated instability of MeCP2 and reduced DNA methylation during active synaptic maturation may disrupt synaptic circuit maturation and cause neurobehavioral abnormalities, as found in animal models of fetal alcohol spectrum disorders (FASDs) [59].

Cannabidiol (CBD), a nonpsychotomimetic component of the Cannabis sativa plant, exhibits therapeutic potential in several psychiatric disorders, including schizophrenia. In the prepulse inhibition animal model, CBD-attenuated MK-801, an uncompetitive antagonist of the N-Methyl-D-aspartate (NMDA) receptor, enhanced DNA methylation [104]. These findings indicate that the antipsychotic effects of CBD involve DNA methylation mechanisms in the ventral striatum. The exposure of mice to CBD orally for 2 weeks caused global DNA hypomethylation, including hypomethylation of the de novo methyltransferase DNMT3A and >3000 additional differentially methylated loci enriched for genes [105] involved in the neuronal function and synaptic structure [106]. The effect of CBD hypomethylation on DNMT3A is significant, as the expression of this de novo methyltransferase in PFC has been shown to cause anxiety-like behaviors in adult mice [106]. Together, these findings may suggest that activation of cannabinoid receptors through cannabinoid abuse, specifically during a stage at which the brain is most vulnerable, alters gene expression via DNA methylation.

## 3. Role of Post-Translational Modification of DNA-Associated Histone Proteins: Cannabinoid Receptors

Histones are proteins that have an essential structural and functional significance in the transition between active and inactive states in chromatin and are responsible for gene regulation and epigenetic silencing [107,108]. The chromatin organization involves two copies of each of the histone H2A, H2B, H3, and H4 proteins, forming a central structured globular domain with a close connection with the DNA [109,110] and a less well-structured amino-terminal tail domain [111,112]. Furthermore, due to the histone fold domain and N-terminal tails, histones are vulnerable to PTMs, such as acetylation, methylation, phosphorylation, and sumoylation [113]. The primary enzymes involved in these PTMs are histone acetyltransferases (HATs), histone lysine deacetylases (HDACs; for example, HDAC-1, HDAC-2, HDAC-3), histone methyltransferase (HMT; for example, G9a, Suv39h1), and histone demethylase (HMD). These PTM-dependent chromatin changes promote the recruitment of DNA-binding proteins, causing a loose or compact chromatin structure at particular genetic loci, which leads to the expression or suppression of a particular gene [113]. Fundamental aspects of different histone modifications have been described in recent reviews [59,62,114].

For the first time, Börner and collaborators demonstrated that exposure to trichostatin A, an HDAC inhibitor, in human Jurkat T cells could regulate *CNR1* expression, where the expression of CB_1_ protein was absent [79]. Reduced expression of *Cnr1* in the cingulate cortex of mice with chronic unpredictable stress was associated with decreased levels of histone H3K9 acetylation (H3K9ac) but not H4K8ac with the *Cnr1* gene [115]. In the FASD study, it was demonstrated that transcriptional activation of Cnr1 followed by widespread neurodegeneration in the PD-7 alcohol-exposed neonatal brain was due to increased H4K8 acetylation (associated with active transcription) and reduced H3K9 demethylation (correlated with transcriptional silencing) at the *Cnr1* gene promoter region [116]. The epigenetic activation of CB_1_ by PD-7 alcohol exposure was associated with enhanced HDAC-1, HDAC-2, and HDAC-3 gene expression [117]. These events, in turn, suppress the expression of synaptic plasticity-related genes such as *Bdnf*, *c-fos*, *Egr1*, and *Arc* [117]. Further studies indicated enhanced enrichment of HDAC-1, HDAC-2, and HDAC-3 at the *Egr1* and *Arc* gene promoter regions. Preadministration of a CB_1_ receptor antagonist (SR141716A) before PD-7 alcohol exposure prevented enrichment of HDACs at the *Egr1* and *Arc* gene promoters and prevented behavioral abnormalities associated with PD-7 alcohol exposure [117]. These observations strongly support the significance of specific histone PTMs’ influence on CB_1_- and CB_1_-mediated functions.

### 3.1. Histone Modifications That Modulate Cnr1 Gene Expression

CB_1_ has been shown to be expressed in the dorsal root ganglion (DRG) and to contribute to the analgesic properties of cannabinoids. In an animal model of neuropathic pain, enhanced enrichment of H3K9me2, a G9a (HMT)-catalyzed repressive histone mark, was found in the promoter regions of the *Cnr1* genes [118]. Furthermore, G9a inhibition in nerve-injured animals not only enhanced CB_1_ expression in DRG but also potentiated the analgesic effect of a CB_1_ agonist on nerve injury-induced pain hypersensitivity [118]. Furthermore, in animals lacking G9a in DRG neurons, nerve injury did not reduce CB_1_ expression in DRG. Additionally, the CB_1_ agonist failed to produce analgesic effects. In addition, nerve injury weakened the inhibitory effect of the CB_1_ agonist on synaptic glutamate release from primary afferent nerves to spinal cord dorsal horn neurons in WT mice but not in DRG neuron-specific G9a-null mice [118]. These observations suggest the function of G9a-mediated histone methylation in the expression of CB_1_ and the analgesic effect of CB_1_. Cocaine self-administration significantly increased *Cnr1* gene expression in NAc, the dorsal striatum (DS), and HP [119]. However, additional studies indicated no enrichment of H3K4me3 and H3K27ac marks [119], two marks usually found at active promoters [120,121]. Additionally, mice exposed to chronic unpredictable stress have been shown to have impaired emotional and nociceptive behaviors and to exhibit reduced CB_1_ expression in the cingulate cortex. Epigenetic evaluation indicated enhanced HDAC-2 and reduced levels of H3K9ac at the *Cnr1* gene in the cingulate cortex compared to controls [115]. It is conceivable that other marks, such as H3K9me2, H3K8ac, or H3K14ac marks, may be altered as found in other conditions and deserve future investigation. Nevertheless, these observations strongly support the consequence of particular histone acetylation or methylation marks on CB_1_ expression and CB_1_-mediated functions.

### 3.2. Histone Modifications That Modulate Cnr2 Gene Expression

CB_2_ is largely expressed in immune cells, and CB_2_ agonists have no analgesic effect [122]. Therefore, inhibition of inflammation by CB_2_ agonists is believed to contribute to the relief of associated pain [123]. Nevertheless, nerve injury enhances CB_2_ expression in DRG, and CB_2_ agonists reduce neuropathic pain [115]. Epigenetic analysis indicated increased enrichment of H3K4me3 and H3K9ac (gene-activating histone marks) and reduced enrichment of H3K9me2 and H3K27me3 (repressive histone marks) at the *Cnr2* promoter in DRG [115]. These findings indicate that nerve injury associated with CB_2_ expression involves specific histone acetylation or methylation mechanisms.

### 3.3. Modulation of Histone Acetylation and Methylation by CB_1_ and CB_2_ Activities

Studies where lymph node cells of mice immunized with a superantigen were exposed to THC showed associations of active histone modification signals with Th2 cytokine genes and suppressive modification signals with Th1 cytokine genes, suggesting that such a mechanism may play a significant role in the THC-mediated switch from Th1 to Th2 responses [124]. These studies suggest that some THC regulation of immune responses involves epigenetic pathways. THC exposure in adolescents transiently enhanced H3K9me3 in PFC by increasing the expression of Suv39H1, a histone lysine methyltransferase, but not G9a, and reduced the expression of the *Homer1*, *Mgll*, *Abat*, and *Dlg4* genes, which are closely associated with synaptic plasticity [125]. Further epigenetic analysis indicated increased enrichment of H3K9me3 at the *Homer1*, *Mgll*, *Abat*, and *Dlg4* genes but not at *Abat*. In the same study, simultaneous inhibition of Suv39h1 and G9a significantly rescued THC-increased H3K9me3 levels [125]. These observations suggest that adolescent THC-induced cognitive deficits involve specific histone methylation enzymes such as Suv39h1 and G9a. In another study, chronic THC administration significantly transiently enhanced H3K14ac and H3K9me2 levels in HP and NAc [125]. However, in the amygdala, these histone modifications are differentially altered by THC [125].

Low-dose THC administration in mature (12 months old) and old mice (18 months) improved the expression of synaptic plasticity-related proteins (synapsin I, synaptophysin, PSD95, pCREB, pERK), including the Klotho and Bdnf gene in HP and cognitive performance [126]. In addition, these changes were associated with enhanced global H3K9ac and H4K12ac and reduced H3K9me3 levels in HP. Furthermore, there is enhanced enrichment of H3K9ac at the Klotho and Bdnf promoter regions. HAT inhibitor treatment blocked the effects of THC on cognitive function and H3K9ac levels, synapsin 1, Klotho, and Bdnf expression. Consistent with HAT inhibitor effects, glutamatergic neuron-specific CB_1_-null mice also prevented THC effects on cognitive function and H3K9ac levels, synapsin 1, Klotho, and Bdnf expression [126]. These findings suggest that histone acetylation changes via CB_1_ signaling in forebrain glutamatergic neurons mediate the beneficial effects of low-dose THC.

CBD has been shown to modify histone marks in different model systems. For example, CBD (10 mg/kg, i.p.) showed enhanced enrichment of H3K4me3 in the FoxA1 binding motif [127]. In the same study, CBD enhanced H3K4me3 and reduced H3K27me3 at specific genes, such as *IL-4*, *IL-5*, and *IL-13*, in splenic CD4+ T cells [127]. In another study, repeated coadministration of CBD and THC (at a 5:1 CBD/THC ratio, i.e., 50 mg/kg/10 mg/kg, i.p. for 15 days), but not the administration of either compound alone, increased H3K9ac and H3K14ac levels, gene-activating histone acetylation marks in the ventral tegmental area of adult male mice [128]. However, similar to DNA methylation, histone modifications in response to exogenous stimuli can vary from tissue to tissue and different brain regions. Consistent with this notion, a study reported differential modifications of H3K4me3, H3K9ac, H3K9me2, H3K27me3, and H3K36me2 in the cerebral cortex, HTM, and pons following systemic administration of CBD (20 mg/kg, i.p.) to adult rats [129]. In the cerebral cortex, CBD enhanced H3K4me3, H3K9ac, and H3K27me3 levels without having any significant influence on H3K9me2 or H3K36me2 levels [129]. In HTM, CBD reduced H3K9ac levels without having any significant influence on H3K4me3, H3K9me2, H3K27me3, and H3K36me2 levels [129]. Last, in the pons, CBD decreased H3K4me3 levels without altering H3K9ac, H3K9me2, H3K27me3, or H3K36me2 marks [129]. The histone modifications induced by CBD were associated with anxiety-related behavioral changes in distinct preclinical studies. For instance, stress duration impacts H3K4, H3K9, and H3K27 methylation levels in HP [130]. In particular, acute stress increased H3K9me3 and reduced H3K27me3 and H3K9me1 levels in HP [130]. In contrast to these results, a week of restraint stress increased H3K9me3 and reduced H3K27me3 and H3K4me3 levels in the same region [130]. Therefore, it is possible that the observed stress-induced reductions in H3K27me3 and H3K4me3 in HP following a week of restraint stress [130] may be reversed by CBD’s enhancing effects on these histone modifications, as observed in the cerebral cortex [129]. Another intriguing anxiety-related epigenetic marker is H3K9ac, and its levels are enhanced in the cerebral cortex by CBD [130]. Additionally, low H3K9ac levels in the central amygdala were suggested to contribute to the maintenance of chronic anxiety and pain [131]. Low-level maternal care-induced anxiety-related behavior in adulthood was associated with reduced H3K9ac levels at the glucocorticoid receptor gene (*Nr3c1*) in rats [132]. Therefore, in the future, more systematic studies are warranted to examine the link between the protective function of CBD on histone modifications at key genes in health and disease (Table 2).

## 4. Regulation of microRNAs and Cannabinoid Receptors

MicroRNAs (miRNAs) are small, noncoding RNAs that function as essential epigenetic regulators of gene expression [134]. MiRNAs accomplish their post-transcriptional regulatory functions by interacting with the 3′ untranslated regions (3′ UTRs), 5′ UTRs, coding sequences, and gene promoters of target mRNAs and cause mRNA degradation, leading to translational repression [134,135]. Furthermore, miRNA interactions with their target genes are dynamic and dependent on their subcellular localization, miRNA and mRNA abundances, and miRNA–mRNA interaction affinity [136]. Thus, they can regulate the expression of networks of genes and entire pathways and are considered master regulators of gene expression [137]. MiRNAs are secreted into extracellular fluids and function as signaling molecules in the form of vesicles, such as exosomes, and mediate cell communication [138,139,140]. Furthermore, abnormal miRNA expression is associated with many human disorders [141,142,143]. Recent articles have simplified the canonical and noncanonical miRNA biogenesis pathways and mechanisms underlying miRNA-mediated gene regulation [136,144]. Moreover, the current knowledge of the miRNA secretion, transfer, and uptake of extracellular miRNAs and their functions has been reviewed elsewhere [145,146]. Additionally, miRNAs are indispensable for the maturation and functioning of the adult brain. Undeniably, several studies have demonstrated the participation of different miRNAs in a wide range of cellular homeostatic processes, including cellular differentiation, development, neural patterning, and synaptic plasticity [147,148,149,150]. Genetic deletion studies have demonstrated that miR-124, miR-125b, miR-132, miR-134, miR-137, and miR-138 control dendritic branching and synaptic maturation (for reference, see [151]). The interference of miRNA biogenesis pathways, such as Dicer, which controls the expression of all miRNAs, indicated the miRNA functions in cell differentiation, neuronal size, dendritic branching, and axonal guidance [152]. These studies ultimately indicated the functions of miRNAs in inhibitory synaptic transmission and cognitive function [153]. Thus, an understanding of the regulatory mechanisms that control the patterns and activity of cannabinoid receptors by miRNA expression has the potential to identify a likely mechanism for cannabinoid receptor expression and mediated function. Therefore, we provide an overview of miRNAs that regulate cannabinoid receptor expression and how the modulation of cannabinoid receptors influences miRNA expression.

### 4.1. miRNAs That Modulate Cnr1 Gene Expression

As discussed above, the cannabinoid system is regulated by and regulates epigenetic mechanisms, though the interactions between cannabinoid receptor modulation and miRNAs are under investigated. Cannabinoids have been shown to drive anticancer effects through miRNA modulation. However, whether ECS promotes tumor growth and progression through miRNA is still unclear. Furthermore, miRNAs have been shown to regulate ECS, promoting or inhibiting cancer growth and progression. For instance, miRNA-1273g-3p promotes the proliferation, migration, and invasion of human colon cancer cells by targeting CB_1_ [154]. In addition, an inverse association was found between CB_1_ and hsa-miRNA-29b-3, indicating that CB_1_ and hsa-miRNA-29b-3 may crosstalk in pediatric low-grade glioma [155]. These findings suggest that miRNAs may interact with cannabinoid receptors to regulate their function, such as in cancer. However, future studies must establish their interactions in various cancers.

The combined computational and experimental evidence indicates that miR-494 controls CB_1_ expression in myocardial cells [156]. There was also an association between miRNA let-7d and CB_1_ expression in several neuronal models [157]. In a spinal cord injury model, miR-338-5p has been shown to target Cnr1, and overexpression of miR-338-5p reduced Cnr1 and provided neuroprotection after spinal cord injury [158]. Because cannabinoids exhibit anti-inflammatory responses, several studies have explored the regulation of miRNAs and their subsequent effects. For example, in obese mice, inhibition of CB_1_ with the specific antagonist AM251 resulted in increased miR-30e-5p and reduced adipocyte storage [159]. MiRNA-30b has been shown to bind to Cnr1 and reduce its expression in cells transfected with miR-30b mimic [160] (Table 3). However, future studies must identify novel miRNAs that may have a direct role in CB_1_ expression and establish their interactions in various neurobehavioral functions.

### 4.2. miRNAs That Modulate Cnr2 Gene Expression

Studies on Cnr2 regulation by miRNA are limited. MiR-187-3p was found to target the 3′ untranslated region (UTR) of the CNR2 gene and inhibit CNR2 expression and differentiation of human osteoblastic precursor cells [161]. The combined computational and experimental evidence suggests that miR-665 controls CB_2_ expression in myocardial cells [156] (Table 3). These findings support the existence of miRNA-binding regions in the Cnr2 gene and their regulatory role in Cnr2 expression. However, additional studies are warranted to examine whether differential regulation of CB_2_ expression is controlled by different miRNAs in health and pathological conditions.

### 4.3. Modulation of miRNAs by CB_1_ and CB_2_ Activities

THC, which acts through CB_1_ and CB_2_ receptors, has been shown to induce functional myeloid-derived suppressor cells (MDSCs) in vivo. In these studies, cells exhibited several differentially expressed miRNAs. Among these miRNAs, miRNA-690 was found to be highly overexpressed in THC-induced MDSCs. These studies suggested that miR-690-targeting genes are involved in myeloid expansion and differentiation and, therefore, in THC immunosuppression effects [163]. AEA anti-inflammatory action significantly involves interleukin 10 (IL-10) induction in the draining lymph nodes (LNs). This function of AEA was associated with miRNAs that target proinflammatory pathways [164]. THC administration before and after simian immunodeficiency virus (SIV) inoculation ameliorated disease progression. In addition, it reduced inflammation in male rhesus macaques. This neuroprotection likely involves miRNAs that regulate the mRNAs of proteins involved in neurotrophin signaling, MAPK signaling, the cell cycle, and the immune response in the striatum of SIV-infected macaques [165]. The anti-inflammatory property of THC was shown to involve an miRNA cluster, specifically miRNA-18a. miRNA-18a is a target of Pten (phosphatase and tensin homolog, an inhibitor of the PI3K/Akt signaling pathway) and is known to suppress T-regulatory cells [166]. THC treatment inhibited the individual miRNAs in the cluster, reversed the effects of staphylococcal enterotoxin B (SEB) on mortality, and alleviated symptoms of toxic shock [166]. Additionally, THC reduced intestinal inflammation in mouse colitis models and SIV-infected rhesus macaques [167]. This neuroprotective function of THC was associated with selective enhancement of miR-10a, miR-24, miR-99b, miR-145, miR-149, and miR-187 expression, which have been shown to target proinflammatory molecules [167]. The combined administration of THC and CBD augmented murine experimental autoimmune encephalomyelitis (EAE) by reducing neuroinflammation and suppressing Th17 and Th1 cells in a CB_1_- and CB_2_-dependent manner. In the same model, studies indicated reduced miR-21a-5p, miR-31-5p, miR-122-5p, miR-146a-5p, miR-150-5p, miR-155-5p, and miR-27b-5p expression while enhancing miR-706-5p and miR-7116 expression [168]. CBD alone was shown to suppress inflammation in an animal model of EAE by modulating several miRNAs [127]. It was shown that prenatal THC exposure enhanced miR-122-5p; reduced the expression of its target, insulin-like growth factor 1 receptor (Igf1r), in adult rat ovary follicular cells; and caused follicular apoptosis [169]. In another study, activation of CB_2_ by a specific agonist (AM1241) was suggested to protect dopaminergic neurons in Parkinson’s disease animals. This function was associated with increased expression of miR-133b-3p and reduced expression of target genes such as *Xist* and *Pitx3* [162]. These studies suggested that the protective function of cannabinoids involves miRNAs. Future studies establishing insight into the complex interactions between miRNA and mRNA in various cannabinoid actions may aid in untangling the molecular underpinnings of the medicinal value of marijuana.

## 5. Conclusions

**Figure 1:**
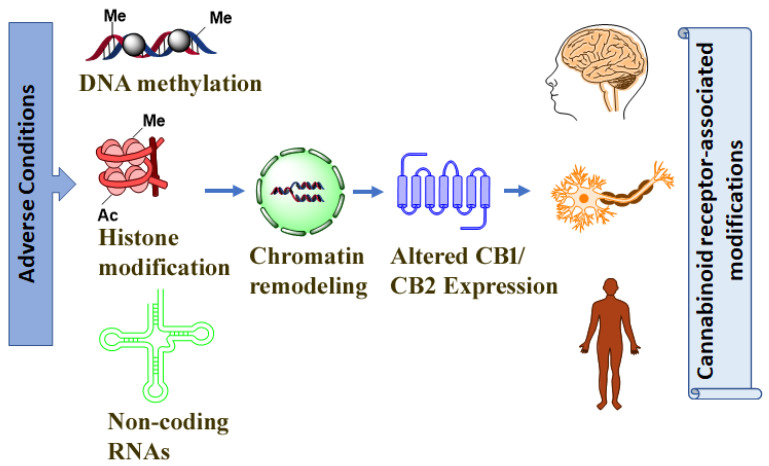
Epigenetic mechanisms epitomize important gene–environment relation mediators behind many human disorders’ pathogenesis. Various adverse conditions such as drug abuse, including alcohol, cannabinoids, and adverse developmental conditions may potentially affect the expression of genes such as cannabinoid receptors, causing the altered functions, at least through epigenetic modifications.

Cannabinoid receptors have been shown to function in health and in a number of neuropsychiatric diseases and pathological conditions in response to various insults. In the current preclinical review, we presented an overview of cannabinoid receptor gene structure that offers potential targets for epigenetic modifications in behavioral and neuropharmacological studies that evaluate cannabinoid receptor expression and epigenetic changes. In addition, we outlined evidence suggesting that the activation and inhibition of cannabinoid receptors and their downstream regulation may involve epigenetic mechanisms that include DNA methylation, histone modifications, and the regulation of miRNA expression. Collectively, these studies support the continued evaluation of cannabinoid receptor regulation by epigenetic modifications and can be potential targets in the treatment of cannabinoid receptor-mediated behavioral and pathological conditions (Figure 1). However, future studies are still warranted to evaluate the direct link between cannabinoid receptor activities and epigenetic mechanisms of action. Thus, epigenetic changes are promising targets for the future development of potential therapeutic agents to treat altered cannabinoid receptors functions.

## Figures and Tables

**Table 1:** DNA methylation and CB_1_ receptor gene (Cnr1) expression.

Model	Treatment/Exposure	DNA Methylation at Cnr1 Promoter	Cnr1 Gene Expression	Reference
Colon cancer specimens	-	↑	↓	[75]
Human epithelial colon cell line LS-174T	Prostaglandin E2	↑	↓	[76]
Rodents (PD 1 to 14)	Maternal separation	↑	-	[77]
Jurkat cells	5-Aza-dC	↓	-	[79]
Humans	THC	↑	↓	[80]
Human colon cancer cells and rats	Extra-virgin olive oil (EVOO). Phenolic extracts (OPE) Hydroxytyrosol (HT)	↓	↑	[81]
Rats L6-S2 (DRG)	Chronic stress	↑	↑	[82]
Mice (PD 7)	Alcohol	↓	↓	[87] [88]
Schizophrenic patients	-	↓	↑	[83]
Rat (PFC)	Methylazoxymethanol acetate exposure	↓	↑	[83]
Human blood mononuclear cells from younger (<30 years old) human obese subjects	THC/alcohol	-	↑	[84]
Rat model	Anorexia	↑	↓	[85]
Schizophrenia patients	-	↑	↓	[36]

ND, not determined.

**Table 2:** Histone modifications at the CB_1_ receptor gene (Cnr1).

Model	Treatment	Histone Modifications	Gene Promoter	Reference
Mice (Cingulate cortex)	Chronic unpredictable stress	-Increased HDAC-2 -Decreased levels H3K9ac	*Cnr1*	[115]
PD-7 Mice	Alcohol	-Increased H4K8ac -Decreased H3K9me2	*Cnr1*	[116]
PD-7 Mice	Alcohol	-Increased HDAC-1, HDAC-2, and HDAC-3	*Egr1*, *Arc*	[117]
Rats	neuropathic pain	-Increased H3K9me2	*Cnr1*	[118]
Male Rats	Cocaine self-administration	-Increased H3K4me3, H3K27ac	*Cnr1*	[119,125]
Female adolescent rats	THC	-Increased H3K9me3 -Increased Suv39H1, histone lysine methyltransferase,	ND	[125]
Female adolescent rats	THC	-Increased H3K14ac and H3K9me2	ND	[125,133]
Mice	THC	Increased global H3K9ac, H4K12ac and reduced H3K9me3	Klotho and Bdnf	[126]
	THC and HAT inhibitor	ND	Decreased H3K9ac, synapsin 1, Klotho, and Bdnf expression	
CD4+ T cells	CBD	Increased H3K4me3 and reduced H3K27me3	IL-4, IL-5, and IL-13	[127]
Adult male mice	CBD and THC	Increased H3K9ac and H3K14ac levels	ND	[128]
Adult rats Cerebral cortex Hypothalamus Pons	CBD	-Increased H3K4me3, H3K9ac, and H3K27me3 levels -Decreased H3K9ac levels -Decreased H3K4me3 levels	ND	[129]
Rats	Acute stress	-Increased H3K9me3 -Decreased H3K27me3 and H3K9me1 levels	ND	[130]
	Chronic stress	-Increased H3K9me3 and decreased H3K27me3 and H3K4me3 levels		
	CBD	-Increased H3K9ac levels		
Prolonged exposure to corticosteroids	Chronic anxiety and pain	-H3K9ac levels in the central amygdala	ND	[131]
Rats	Maternal care-induced anxiety-related behavior	-Decreased H3K9ac levels	glucocorticoid receptor gene (Nr3c1)	[132]

ND, not determined.

**Table 3:** miRNA changes related to cannabinoid receptor gene expression.

miRNA	Experimental Model	Target/Gene Expression	Reference
miRNA-1273g-3p	Human colon cancer cells	CB_1_	[154]
hsa-miRNA-29b-3	Low-grade glioma		[155]
miR-494	Myocardial cells		[156]
miRNA let-7d	SH-SY5Y neuroblastoma cells Zebrafish, mice (cortex, striatum, and hippocampus) primary striatal neurons		[157]
miR-30e-5p	Mice (obese model)		[159]
miR-338-5p	Rats (spinal cord injury)	Cnr1	[158]
MiRNA-30b	Rat models/cell models		[160]
MiR-187-3p	Human osteoblastic precursor cells	Cnr2	[161]
miR-133b-3p	PD mouse model	Xist and Pitx3	[162]
miR-665	Human (myocardial cells)	CB_2_ and Cnr_2_	[156]
miRNA-690	Mouse (MDSCs)	CB_1_ and CB_2_	[163]
